# Hearing Function and Nutritional Status in Aviation Pilots from Spain Exposed to High Acoustic Damage

**DOI:** 10.3390/nu14204321

**Published:** 2022-10-15

**Authors:** Carmen Morais-Moreno, Ana M. Montero-Bravo, Ana M. Puga, Mª de Lourdes Samaniego-Vaesken, Mar Ruperto, Rocío Marco Mendez, Álvaro Vicente-Arche, Gregorio Varela-Moreiras, Teresa Partearroyo

**Affiliations:** 1 Nutrition for Life, ref: E02/0720, Departamento de Ciencias Farmacéuticas y de la Salud, Faculty de Farmacia, Universidad San Pablo-CEU, CEU Universities, 28660 Boadilla del Monte, Spain; 2 Centro de Instrucción de Medicina Aeroespacial (CIMA), 28850 Madrid, Spain

**Keywords:** homocysteine, folate, hearing loss, aviation, pilots

## Abstract

Noise-induced hearing loss is the most frequent and preventable occupational disease. Aviation pilots are a vulnerable population, as they spend many hours exposed to noise pollution in their working environment. Different studies suggest that certain dietary compounds may play a key role in the etiology and prevention of this pathology. We aimed to study the relationship linking auditory function, dietary intake, and some serum biomarkers in pilots, exposed to noise in the work environment. A total of 235 pilots, who undergo a periodic medical examination at the Centro de Instrucción de Medicina Aeroespacial (Madrid, SPAIN), were evaluated. Auditory function was assessed by tonal audiometry. Energy and nutrient intakes were estimated by 24 h recall (DIAL^TM^ program). Serum homocysteine (Hcy) was determined in a Cobas 6000^TM^ multi-analyzer while folate, vitamin B_12_, and D were analyzed in a Cobas e411^TM^. The prevalence of hearing loss (HL) was 64.3%. HL was significantly related to age (*r =* 0.588, *p* ≤ 0.001) and flight hours (*r =* 0.283, *p* ≤ 0.001). A multiple linear regression model was used to assess the relationship among HL, flight hours, serum folate, and Hcy serum levels. HL was significantly (*p* < 0.050) associated with flight hours (**β** = 0.246), serum folate (**β** = −0.143), and serum Hcy (**β** = 0.227). Nutritional interventions would be of great interest to monitor and slow down the HL progression in populations exposed to noise pollution in their workplace.

## 1. Introduction

Noise-induced hearing loss (NIHL) represents a major occupational health hazard worldwide [1,2] as it is considered the most frequent and preventable occupational disease [3] for which there is no effective treatment available yet. According to the World Health Organization (WHO) [4], it is estimated that over 1.5 billion people globally live with hearing loss (HL), and approximately 11% of the world’s adult population works in a noisy environment with the involved risk [5]. However, recreational activities also accelerate such loss. In fact, 1.1 billion young people (12–35 years) worldwide are exposed to excessive noise [4].

HL has a multifactorial origin [6,7], including both genetic and environmental factors. Among the former, mutations in genes or their regulatory elements involved in the development, structure, or function of the ear stand out, while environmental factors include treatment with ototoxic drugs, nutritional deficiencies, aging, and especially, exposure to noise [1,8,9,10,11]. These last two factors when combined represent the main contributors to HL. Natural aging causes a type of HL called presbycusis which usually begins around the age of 60–65 years when hearing thresholds at high frequencies start to increase [12]. However, presbycusis may be accelerated by excessive exposure to intense noise, becoming evident in the 40s, and can be comparable to that of a 60–90-year-old [13,14,15].

Aviation pilots from both military and civil crews are a population with special characteristics due to their continuous state of alertness during the flight, stressful situations during takeoff and landing maneuvers, continuous shifting schedules, alterations of circadian rhythm, and pressure changes, which, together with the limited meal options during flight and the number of hours seated in the cockpit, lead to a frequently inadequate lifestyle [16]. Because of all these factors, this population showed an increased risk of cardiovascular diseases, cancer, neurologic, and immunologic disorders [17]. Pilots are also a vulnerable population to NIHL as they spend many hours exposed to noise pollution in their working environment, which triggers different noise-induced hearing pathologies, such as tinnitus or acoustic trauma [18,19]. It is well known that noise exposure in the workplace must be always monitored, not exceeding 85 decibels (dB) during 8 h in a row [20]. As decibels increase, the exposure time to produce hearing damage decreases so that a few seconds of exposure to 120–140 dB is sufficient to cause irreversible hearing damage. However, during takeoff maneuvers, 150 dB may be reached inside an aircraft, with a consequent hearing health risk if appropriate personal protective devices are not used [21,22].

Several epidemiological studies have shown an association between deficiencies of different nutrients and the progression of HL [13,14,15]. Concretely, insufficient folate levels have been correlated with the onset of HL, coinciding with low vitamin B_12_ concentrations and/or high serum homocysteine (Hcy) levels [13,14,15,23,24]. Along these lines, other studies have provided evidence of a potential protective effect of dietary supplementation with folic acid (FA) [25].

On the basis of the HL studies mentioned above, attempts have been made to lower systemic Hcy levels in several human supplementation studies [25,26,27]. In addition, two studies from our research group using mouse models have identified a link among Hcy metabolism, HL, and folate deficiency [26,27,28]. Thus, we have showed that folate deficiency induces premature HL in animals of different genotypes, leading to alterations in the cochlear structure that correlate with changes in cochlear Hcy metabolism, associated with oxidative stress and increased levels of protein homocysteinylation [28]. In addition, a long-term omega-3 fatty acid rich-diet seems also to be associated with a lower risk of HL [29,30].

Considering all the above, in the present work, we aimed to determine the prevalence of HL and its relationship with nutritional status in a sample of aviation pilots from Spain exposed to noise pollution in the work environment.

## 2. Materials and Methods

A descriptive cross-sectional observational study was conducted from November 2019 to February 2021. A final sample of 235 Spanish aviation pilots aged 22–65 years, who undergo a periodic medical examination at the Centro de Instrucción de Medicina Aeroespacial (Madrid, SPAIN), was analyzed. Recruitment of volunteers was performed through an informative session with potential participants. Inclusion criteria were people aged 18–65 years working as airline pilots in Spain with at least 1 year of minimum work experience.

Exclusion criteria were (a) subjects suffering from pathologies related to cardiovascular diseases, diabetes, and/or cancer, (b) subjects under treatment with drugs related to folate metabolism such as antibiotics, anticonvulsants, barbiturate drugs, antiparasitic agents, immunosuppressants, ototoxic drugs, and/or vitamin or mineral supplements, and (c) subjects who have suffered or suffer at the time of the study from an auditory pathology that compromises their hearing function.

Ethical approval was granted by the Clinical Research Ethics Committee of the CEU San Pablo University (Madrid) with the approval reference 458-20-38. The ethical principles of the Declaration of Helsinki were followed, and all participants’ rights were respected. All volunteers signed an informed consent prior to participation in the study. Data processing was carried out under the Personal Data Protection and Digital Rights Guarantee Organic Law 3/2018.

### 2.1. Study Protocol

At the time of recruitment, after the explanation of the project and the signing of the informed consent, the following tests were carried out:

Hearing evaluation: The auditory function was assessed by tonal audiometry using Madsen Itera II Otometrics equipment^TM^. Audiograms were collected in a soundproof booth to determine the hearing threshold using pure tones at frequencies of 250, 500, 1000, 2000, 3000, 4000, and 8000 Hz increasing by 5 dB [31]. The American Speech–Language–Hearing Association (ASHA) classification was used to determine the degree of HL: normal, <15 dB; slight, 16–25 dB; mild, 26–40 dB; moderate, 41–55 dB; moderately severe, 56–70 dB; severe, 71–90 dB; profound, >91 dB.

Dietary assessment: Data from 3 days (two working days, considered as flying days, and one non-working day or holiday) were collected through 24 h dietary recalls to establish usual daily intake [32], by following the EU menu methodology [33]. The first recall was performed at the time of recruitment. Subsequent recalls were conducted by telephone or via email, with at least a 1 week interval between recalls. Nutrient and energy intakes were estimated using the Dietowin 8.0^TM^ software program (Dietowin SL, Sabadell, Spain).

Biochemical analysis: A single blood extraction after overnight fasting (performed the same day of the first 24 h dietary recall and the audiometry) was obtained by venipuncture. Serum Hcy was determined by the enzymatic method in a Cobas 6000^TM^, while serum folate, vitamin B_12_, and vitamin D were analyzed by chemiluminescence in a Cobas e 411^TM^ immune analyzer.

### 2.2. Statistical Analysis

Results are presented as the mean ± standard deviation. Significant mean differences were obtained through the Student’s *t*-test, considering *p* ≤ 0.05, *p* ≤ 0.01, and *p* ≤ 0.001 as significant. Results were obtained for the total sample and segmenting by age in two groups (<40 years old and ≥40 years old).

The relationship linking HL, dietary intake, and nutritional biochemistry biomarkers was assessed using the Pearson’s correlation coefficient. A multiple linear regression model was used to explain the association between HL as a dependent variable and age, serum folate, and serum Hcy variables as independent predictors. Statistical analysis was performed using IBM SPSS v27.0 Statistics software (IBM Corp., Armonk, NY, USA).

## 3. Results

### 3.1. Sample Description

Initially, a total of 288 volunteers (284 men and four women) were recruited for the study. Women were excluded for not being a representative number, and 49 men were withdrawn from the study after meeting some of the exclusion criteria or not adequately accomplishing a part of the study. The final sample of workers exposed to noise consisted of 235 pilots (men) living in Spain. As shown in Table 1, the mean age was 41.4 ± 11.1 years, with a mean of 4848.9 ± 5390.0 flight hours and 17.5 ± 10.8 years of flying experience. When sorting the sample by age groups, volunteers under 40 years old had a mean age of 30.0 ± 5.2 years, a mean of 1324.8 ± 1270.5 flight hours, and 7.8 ± 4.2 years of flying experience, whereas volunteers over 40 years of age presented a mean age of 49.2 ± 6.3 years, 7282.8 ± 5791.9 flight hours, and 24.3 ± 8.6 years of flying experience.

**Figure 1:**
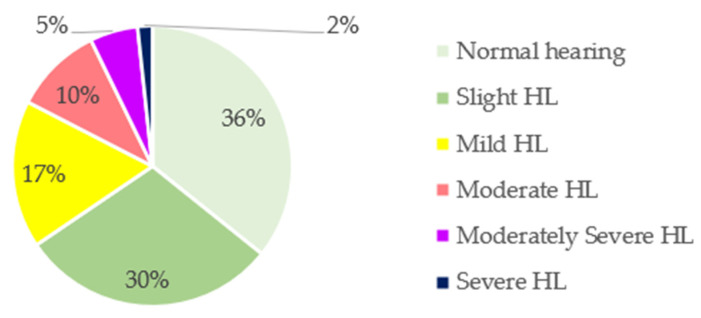
Classification of hearing loss (HL) in the total sample according to the American Speech–Language–Hearing Association (ASHA).

The prevalence of the degree of HL in the total sample is shown in Figure 1. To determine that degree, the ASHA classification was used as a reference: normal, <15 dB; slight, 16–25 dB; mild, 26–40 dB; moderate, 41–55 dB; moderately severe, 56–70 dB; severe, 71–90 dB; profound, >91 dB. Almost two-thirds (64%) of the sample exhibited some degree of HL. Only 36% exhibited normal hearing, whereas 30% showed slight HL, 17% showed mild HL, 10% showed moderate HL, 5% showed moderately severe HL, and 2% showed severe HL.

**Figure 2:**
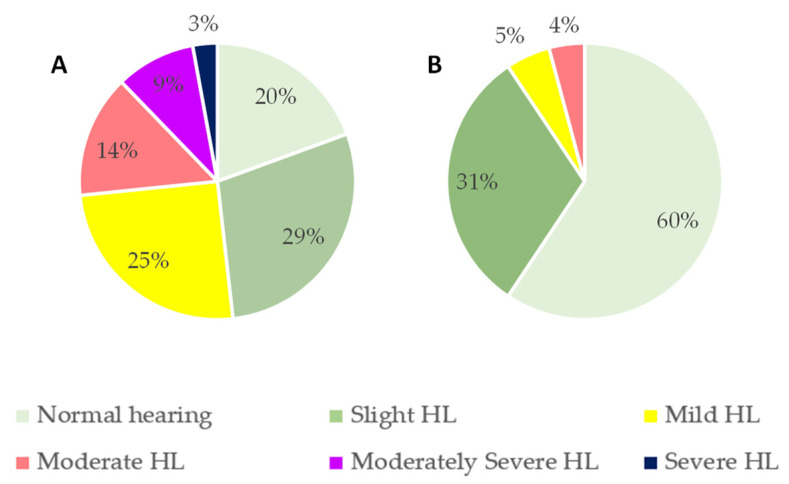
Classification of hearing loss (HL) sorted by age groups according to the American Speech–Language–Hearing Association (ASHA) classification: (**A**) <40 years old; (**B**) ≥40 years old.

Considering the sample by age groups, 40% of volunteers aged 22–39 years exhibited some degree of HL, while this prevalence increased to 80% in volunteers aged 40 years or older (Figure 2). Figure 2B shows severe degrees of HL in the older group when compared to Figure 2A.

**Figure 3:**
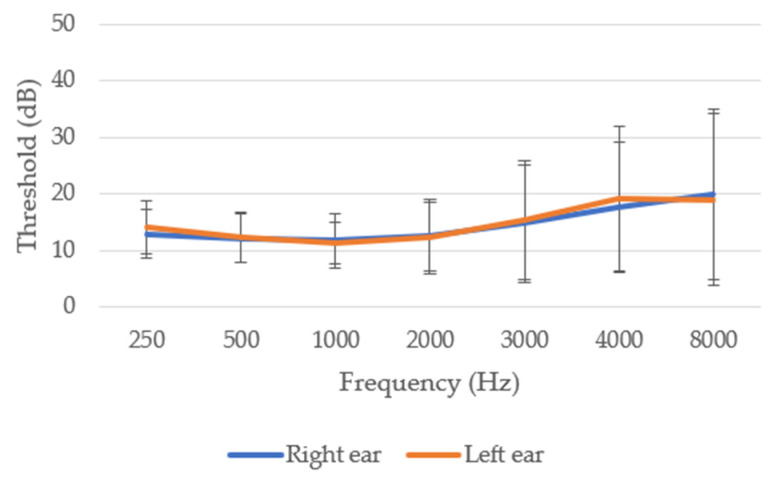
Audiometry of the 235 Spanish aviation pilots studied in the total sample.

Likewise, in line with Figure 3, NIHL begins to be evident between frequencies 1000 and 4000 Hz, related to speech. This HL is accentuated at frequencies 4000–8000 Hz, related to the field of aviation (engines, turbines, etc.). Our data show a clear correlation between HL and age and the number of flight hours, indicating a progressive HL (*r =* 0.588, *p* ≤ 0.001 with age; *r =* 0.283, *p* ≤ 0.001 with flight hours). In addition, a significant positive correlation between right and left ears was obtained (*r =* 0.681; *p* ≤ 0.001).

**Figure 4:**
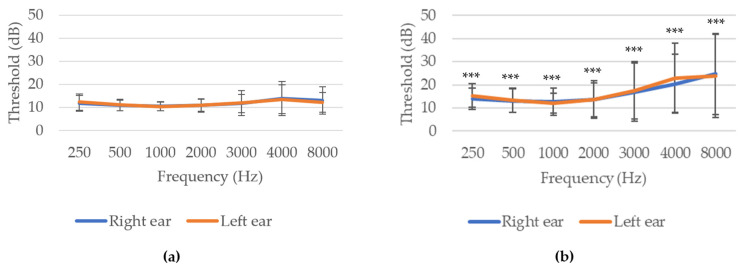
Audiometry of the sample sorted by age groups: (**a**) <40 years old; (**b**) ≥40 years old. *** *p* ≤ 0.001 indicate significant differences between age groups (Student’s *t*-test).

When comparing both age groups, hearing thresholds were significantly (*p ≤* 0.001) higher in volunteers aged 40 years or older compared to those under 40 years. Specifically, HL was greater at higher frequencies (Figure 4). 

Mean daily intakes of energy, macronutrients, alcohol, fiber, and water and their distributions are shown in Table 2 and Table 3. Mean energy intake was 2042.8 ± 449.7 kcal/day, regardless the age. From the total sample, 54.0% had an intake below, 0.4% above, and 45.5% within the recommended intakes according to the European Food Safety Authority (EFSA) [34]. When comparing both age groups, 53.1% of volunteers aged 22–39 years had an intake below, 45.8% within, and 1% above the recommendations while 54.7% of volunteers aged 40–66 years had an intake below and 45.3% within the recommendations [34]. Fiber intake for the total sample was 22.8 ± 8.0 g/day, well below EFSA recommendations for men aged 22–66 years. Mean water intake was 1779.4 ± 490.7 mL/day, below the EFSA recommendation of 2.5 L/day for men. No significant differences between age groups were found, however, in water, monounsaturated fatty acid (MUFA) (g), polyunsaturated fatty acid (PUFA) (g), ω-3 fatty acid, alcohol, and fiber intake. However, energy, protein, carbohydrate, fat, and saturated fatty acid (SFA) (g) intakes were significantly higher in the younger group (*p* ≤ 0.05 for fats, *p* ≤ 0.01 for SFA, and *p* ≤ 0.001 for energy, proteins, and carbohydrates).

In terms of the contribution of macronutrients to the total energy (TE) intake, for the whole population, dietary fats had the highest value (42.2% ± 6.1%), followed by carbohydrates (36.5% ± 6.7%) and proteins (17.5% ± 3.3%), indicative of a very unbalanced energy profile. In fact, when comparing these values with the recommendations, the contribution of fats to TE was above the recommendations while the contribution of carbohydrates to TE was well below the recommendations. Only 21.7% of the participants met the protein recommendations, while 78.3% had a higher intake than recommended. Almost the entire sample (89.9%) did not meet the carbohydrate recommendations, and 87.2% had a fat intake higher than recommended. In terms of the fat profile, volunteers showed an adequate intake of MUFA (19.8% ± 3.9% TE) and PUFA (6.2% ± 2.2% TE), but an excessive intake of SFA (12.4% ± 2.3% TE). Even so, the mean fat quality indices (PUFA/SFA = 0.5 ± 0.3 and (PUFA + MUFA)/SFA = 2.2 ± 0.6) met the Nutritional Objectives of the Spanish Society of Community Nutrition (SENC) [35]. The contribution of carbohydrates was significantly higher (*p* ≤ 0.05) in the younger group, whereas the contribution of fat was significantly greater (*p* ≤ 0.05) in the older age group, albeit not meeting the recommendations in either case. However, no differences were found in the protein percentage between the age groups. Regarding the fat profile, the MUFA and PUFA contributions were both significantly higher (*p* ≤ 0.05) in the older age group while no differences in SFA contribution were found. Alcohol contribution to the TE was 1.6% ± 2.4% of the TE, and no significant differences between both groups were obtained.

Daily intakes of vitamins B_1_, B_2_, B_3_, B_5_, B_6_, B_8_, B_9_ (folates), B_12_, C, A, D, E, and K in the total sample are shown in Table 4. The studied population had adequate intakes of vitamins B_1_, B_2_, B_3_, B_5_, B_6_, B_12_, C, K, and A, but lower intakes of folates and vitamins B_8_, D, and E were observed when compared to the recommended dietary intakes according to the EFSA [34]. It should be noted that only 49.4% of the sample met EFSA’s recommended intakes [34] for folates, and 28.9% of the sample met the recommended intakes for vitamin E. Likewise, 97.0% had potentially inadequate intakes of vitamin D. Intakes of vitamin B_1_ (*p* ≤ 0.05), vitamins B_2_ and B_3_ (*p* ≤ 0.005), vitamins B_5_ and B_6_ *(p* ≤ 0.001), and vitamin B_8_, folates, and vitamin K (*p* ≤ 0.05) were higher in the younger group. On the other hand, no significant differences were found for vitamins B_12_, C, A, D, and E intakes between the age groups.

Table 5 shows the daily intake of several minerals: calcium, iron, iodine, magnesium, zinc, selenium, potassium, and phosphorus. In the studied population, lower intakes of calcium, iodine, magnesium, and zinc were observed compared to EFSA recommended intakes for men aged 22–66 years [34]; 49.8% were below the recommendations for calcium, 78.7% below those for iodine, and 45.5% and 49.4% below the recommended intakes for magnesium and zinc, respectively. However, mean intakes of iron, phosphorus, and selenium were potentially adequate. When comparing both age groups, significant differences were found in calcium, iodine, and phosphorus (*p* ≤ 0.001), as well as in iron and magnesium (*p* ≤ 0.05), being higher in the younger group. No significant differences were obtained for zinc, selenium, and potassium according to the age groups.

### 3.2. Biochemical Parameters

Serum values of Hcy, vitamin B_12_, folate, and vitamin D are shown in Table 6. When comparing the obtained biochemical parameters with the normal laboratory reference values, serum Hcy levels were higher than reference values in the total sample and both age subsamples. Even though serum vitamin B_12_ and folate levels were within the limits, folate levels were in the lower range. Similarly, 25-hydroxyvitamin D levels were below the lower limit in the total sample, as well as in both age groups.

Volunteers over 40 years of age had significantly higher (*p* ≤ 0.001) serum Hcy levels, while, on the contrary, this group exhibited significantly lower vitamin B_12_ values (*p* ≤ 0.05). No significant mean differences were found between both age groups in folates and vitamin D serum parameters. In fact, a positive correlation was obtained between serum folate levels and folate intake (*r* = 0.242, *p* ≤ 0.001). In contrast, no correlations were found between serum levels and vitamin D (*r* = 0.119, *p* = 0.069) and B_12_ (*r* = 0.059, *p* = 0.368).

### 3.3. Hearing Loss and Nutritional Status

A significant positive correlation of the overall HL percentage with age (*r* = 0.588, *p* ≤ 0.001), as well as with flight hours (*r* = 0.283, *p* ≤ 0.001), was found, and several multiple linear regression models were used to assess the relationship among HL, age, and the flight hours to elucidate the real influence of each parameter on hearing status (Table 7 and Table 8). HL was significantly related to age (*p* ≤ 0.001) and flight hours (*p* ≤ 0.001), as shown in Table 7 and Table 8. Therefore, older age and more flight hours were correlated with greater HL.

Likewise, a multiple linear regression model was used to assess the relationship among HL, flight hours, serum folate, and Hcy serum levels. According to the obtained results, HL was significantly related to flight hours (*p* ≤ 0.001), serum folate (*p* = 0.032), and serum Hcy (*p* ≤ 0.001), as shown in Table 9. Consequently, the nutritional status related to the methionine cycle seems to play a remarkable role in HL development associated with noise exposure.

## 4. Discussion

NIHL is the most common occupational disease in Europe and the United States [1,2]. Currently, there is no available treatment for auditory disorder management. Vitamin deficiencies, including folate deficiency, are associated with HL. However, to date, the impact of insufficient nutrient intake on the degree of hearing impairment that noise exposure may cause in Spanish aviation workers has not been assessed. The first step in developing prevention and repair strategies to intervene before the damage becomes irreversible is to better understand the interrelationship between HL and diet. In fact, in our study, in order to try to assess this relationship, a linear regression model among HL, flight hours, serum Hcy, and serum folate levels was obtained. The more serum Hcy and, thus, the lower serum folate levels, together with an increase in flight hours, led to a higher HL in our studied population, which was exposed to noise pollution at their workplace.

Concretely, in Spain, the law that regulates HL in workplace only considers it when the threshold is 25 dB or more [36]. Although, in our study population, the prevalence of mild–severe HL was 34%, an additional 30% had a slight HL according to the ASHA classification. Therefore, one-third of the sample is at risk of developing HL in the short term. It should be noted that the prevalence obtained in this study is higher than that obtained in an Indian Air Force study [37], where a prevalence of 26.2% was found. On the other hand, our value is lower than that obtained in a study carried out in Saudi military pilots where the prevalence was 42% among fixed-wing pilots [38], similar to our volunteers. Several experimental animal models have shown that the onset and progression of HL are closely related to nutrient availability and metabolism [11]. Therefore, reduced folate concentrations have been found in individuals with presbycusis and sudden deafness, which correlate with simultaneously low vitamin B_12_ values [13,14,23] and increased Hcy levels [15,24]. In this regard, we previously found that alterations in Hcy metabolism increase susceptibility to HL [39]. Likewise, similar results were also found in this study, where serum Hcy levels above the upper limit of the reference value converge with serum folate levels at the lower limit of the reference values. This association is more evident in those over 40 years of age who have significantly higher serum Hcy values and significantly lower vitamin B_12_ values than the younger group. In fact, our data show that, although there are no significant differences, vitamin B_12_ intake was higher in the older age group, while these were the volunteers who showed significantly lower serum vitamin B_12_ values. In fact, our data are in line with other studies showing that serum values are within the reference limits until natural aging begins, after which vitamin B_12_ serum values start to decrease. This fact highlights the need to monitor serum vitamin B_12_ levels in this segment of the population [15]. Even though there were no significant differences between groups in folate serum values, folate intake was significantly lower in those over 40 years of age. Moreover, 53.9% of the population exposed to noise pollution at their workplace had inadequate folate intake, which correlated with serum levels. In fact, a study carried out in fighter pilots from the Tyndal Air Force Base in Florida concluded that the US military recommended dietary intakes were met for all micronutrients except for folate (78%) [40]. Our results showed that both intake and serum levels were negatively correlated with HL. Likewise, serum Hcy levels were positively correlated with HL. Therefore, lower folate intake and lower serum folate, together with higher serum Hcy concentrations, are associated with a higher percentage of HL.

As for the energy and macronutrient intake of the studied population, our data showed an inadequate profile. In a study that was carried out on Israeli air force pilots, the average energy intake was 2657 ± 168 kcal/day [41], a higher value than that encountered in a study conducted in Greek pilots (2210.5 kcal) [42]. According to data from the ANIBES (“Anthropometric data, macronutrient and micronutrient intake, practice of physical activity, socioeconomic data, and lifestyles in Spain”) study [43], a benchmark study on intakes in the Spanish population, only 78% of adult men in the Spanish population reached the average energy intake recommended by the EFSA [34]. The mean energy intake value obtained in adults aged 22–66 years was 1816 ± 512 kcal/day, specifically 1966 ± 543 kcal/day in adult men [43], compared to the 2555 kcal/day recommended for a middle-aged man with a moderate level of physical activity. Our results showed a mean value of 2042.8 ± 449.7 kcal/day, a value slightly higher than but very similar to the data obtained in the ANIBES study and well below the value for the Israeli pilots, although it should be noted that the mean age of the Israeli pilots (25.4 ± 1.05 years) was considerably lower than that of the volunteers in our study (41.4 ± 11.1 years). However, in a study [44] carried out in Irani military pilots with a mean age of 42.0 years (similar to our 41.4 year mean age), the mean energy intake was 3106 kcal/day, considerably higher compared to our findings. Only 45.5% of our volunteers had an intake within the recommendations, with this value being considerably lower than in the ANIBES study. Therefore, this population is below the average requirement considering a physical activity level of 1.6 (moderate) for adult men. 

According to the EFSA [34], an adequate energy profile is the one in which 45.0–60.0% of the TE comes from carbohydrates, 15% comes from protein, and 20.0–35.0% comes from fat. In the aforementioned study conducted in fighter pilots from Florida [40], mean percentages of energy derived from carbohydrate, protein, and fat were 48.3%, 16.1%, and 34.2%, respectively, which comply with EFSA recommendations. A study conducted in Greek pilots resulted in a very similar pattern for the caloric profile as 52% from the TE was provided by carbohydrates, and 32% was provided by fats, with only the protein contribution being higher than recommendations (17%) [42]. In the study carried out in Israeli pilots [41], the contribution of macronutrients to TE was 47% for carbohydrates, 17% for proteins, and 36% for fat. Our data showed worse results, not complying with the EFSA recommendations, and resembling more the dietary data of the Spanish general population than those of pilots from other countries. The ANIBES study [43] showed an imbalance in the calorie profile of the Spanish population. Specifically, carbohydrates contributed the highest proportion, followed by fats and proteins. According to our data, carbohydrate intake was 36.5%, well below the recommendations and below the average for the Spanish population. In contrast, protein intake was 17.5%, which is above the recommended upper limit; similarly, fat intake in our study population was 42.2%, higher than the recommendations. In terms of fat profile, MUFA contributed 19.7% of TE, which was close to the recommendations and higher than Israeli pilots (11.4%) and the Spanish population (16.8%) [43]. Similarly, the intake of PUFA was in line with the recommendations (5.6%), even though it was a lower value that that encountered in the Israeli pilot sample (10.6%), but very similar to that found in the Spanish general population (6.6%). However, SFA intake (12.5%) was found to be higher than the recommendations, similar to the ANIBES study (11.7%) and well above the Israeli (9.5%) and the Greek (8%) studies. Nevertheless, fat quality indices used were within the recommendations. 

Fiber intake was low in the studied population (22.8 g/day) compared to EFSA recommendations for men (35 g/day). However, our result was higher than in the Israeli study. A recent study conducted in older adults showed a modest association between fiber intake and tinnitus, which, as indicated before, is one of the most frequent NIHL pathologies among aviation pilots [19]. Fiber intake seems to reduce the risk of tinnitus through the reduction in vascular risk [45], which is linked to the methionine cycle and a hyperhomocysteinemia status [46]. 

With regard to micronutrient intake, we observed a potential insufficiency for magnesium, calcium, zinc, folate, and vitamins D and E. These data are in line with that obtained in the ANIBES study, where the intake of folates and vitamin D was the lowest in Spanish subjects with intakes above 80% the recommended daily Spanish intakes for men [47]. Special attention should be paid to the insufficient intake of vitamin D in this population, where 97.0% of the sample did not meet the recommended intakes. Indeed, serum vitamin D values were found to be below the lower limit of the reference value. Our data also coincide with the Israeli study [41], where lower intakes for calcium, zinc, and magnesium were obtained. When comparing the micronutrient intake, our data show higher intake values of iron and vitamins A and B_12_ than the ones in the Israeli study. Similarly, higher values of vitamin A, D, and B_12_ intakes were found when compared to the Irani military pilot study (607.3 µg, 2.1 µg, and 5.2 µg, respectively) [44]. Contrarily, lower intake values of vitamins C and E and folates were found compared to the Israeli study [41] and the Irani study. In fact, the study in Iranian pilots compared the antioxidant status between pilots and non-flight personnel and concluded that pilots may need more vitamin C as they are exposed to environmental factors such as noise that contribute to a higher oxidative stress level [44].

Oxidative stress is one of the factors triggered by noise exposure that stands behind NIHL [48]. A clinical study on 35 hearing-impaired subjects showed that the total oxidative status and oxidative stress index were increased [48]. Being known as the antioxidant vitamins, vitamins C and E play a key role neutralizing free radicals and reactive oxygen species. Even though there is a lack of studies that relate the relationship between these micronutrients and HL in aviation pilots, antioxidant combinations have been widely used as potential treatment against HL. A study showed that oral supplementation with vitamin E, vitamin C, and *beta*-carotene reduced the subjective discomfort and intensity of tinnitus in patients with idiopathic tinnitus [49], one of the main NIHL pathology aviation pilots usually face. In fact, it appears that the combination of the antioxidant micronutrients such as vitamin C and E with standard therapy is more effective in improving hearing than standard therapy alone due to the synergistic effect, leading to an accumulation of sufficient amounts of micronutrients in the human inner ear [50,51].

## 5. Strengths and Limitations of the Study

The fact that the relationship between HL and nutritional status was studied on the basis of the biochemical parameters and not only on 24 h recalls is undoubtedly one of the main strengths of this study, as it provides more accuracy. Another strength is the number and homogeneity of the volunteers conforming this sample, as they were airline pilots, all subject to the same range of aviation-related frequencies. In addition, it should be noted that few studies have analyzed the dietary intake in airline pilots, let alone the relationship with HL; hence, this study provides innovative data that attempt to bring this relationship together. Lastly, this is a pioneering study in Spain as, to date, there is no research that relates nutritional status with hearing loss in a Spanish population exposed to noise in the work environment, specifically in aviation pilots. One limitation in our study is the under-reporting bias implicit in the 24 h dietary recall questionnaires that were used in this study; in particular, the first 24 h recall was conducted at the time of the medical examination required for the renewal of their flying license. Another fact that should be considered is the paucity of women who were recruited. As they did not represent a significant number, we had to withdraw them. This is since, in the world of aviation, although more and more women are working as pilots, they are still a minority in the aeronautical sector.

## 6. Conclusions

The studied sample exposed to noise pollution in the workplace had a high prevalence of HL, which increased with age and flight hours at higher frequencies. Our results found a clear association between HL and flight hours, serum folate, and serum Hcy levels. In addition, several micronutrients including folate, iodine, and vitamin D intakes were found to be well below the dietary reference intakes. The overall results suggest the strong need for regular monitoring of dietary intake in a vulnerable group such as the one studied, in order to establish a strategy for nutritional interventions.

## Figures and Tables

**Table 1:** Description of the total sample and sorted by age groups.

	Total Sample (*n* = 235)	<40 Years Old (*n* = 96)	≥40 Years Old (*n* = 139)
Age (years)	41.4 ± 11.1	30.0 ± 5.2 ***	49.2 ± 6.3
Flight hours (h)	4848.9 ± 5390.0	1324.8 ± 1270.5 ***	7282.8 ± 5791.9
Flying experience (years)	17.5 ± 10.8	7.8 ± 4.2 ***	24.3 ± 8.6

Results are expressed as the mean ± standard deviation. *** *p* ≤ 0.001 indicate significant differences among subsamples (Student’s *t*-test).

**Table 2:** Daily water, energy, and macronutrient intake in the total sample.

	Total Sample (*n* = 235)	<40 Years Old (*n* = 96)	≥40 Years Old (*n* = 139)
Water intake (mL)	1779.4 ± 490.7	1834.9 ± 432.9	1741.0 ± 524.9
Energy (kcal)	2042.8 ± 449.7	2184.8 ± 454.3 ***	1944.8 ± 420.8
Proteins (g)	88.8 ± 23.1	95.2 ± 23.7 ***	84.4 ± 21.7
Carbohydrates (g)	186.6 ± 53.3	204.5 ± 51.9 ***	174.3 ± 50.8
Fats (g)	95.9 ± 25.9	100.7 ± 27.3 *	92.5 ± 24.6
SFA (g)	28.4 ± 9.3	30.6 ± 10.3 **	26.9 ± 8.2
MUFA (g)	44.2 ± 12.8	46.5 ± 13.0	43.7 ± 12.5
PUFA (g)	13.9 ± 5.7	14.2 ± 5.5	13.9 ± 5.8
ω-3 FA (g)	2.2 ± 1.2	2.2 ± 1.2	2.2 ± 1.2
Alcohol (g)	4.7 ± 7.0	4.7 ± 7.5	4.7 ± 6.7
Fiber (g)	22.8 ± 8.0	23.7 ± 8.3	22.2 ± 7.8

Results are expressed as the mean ± standard deviation. SFA: saturated fatty acids; MUFA: monounsaturated fatty acids; PUFA: polyunsaturated fatty acids. * *p* ≤ 0.05, ** *p* ≤ 0.01, and *** *p* ≤ 0.001 indicate significant differences between groups (Student’s *t*-test).

**Table 3:** Contribution of macronutrients to the total energy and dietary fat quality indices.

	Total Sample (*n* = 235)	<40 Years Old (*n* = 96)	≥40 Years Old (*n* = 139)
Proteins (%)	17.5 ± 3.3	17.5 ± 3.2	17.5 ± 3.5
Carbohydrates (%)	36.5 ± 6.7	37.6 ± 6.2 *	35.7 ± 6.9
Fats (%)	42.2 ± 6.1	41.3 ± 5.5 *	42.9 ± 6.5
Alcohol (%)	1.6 ± 2.4	1.5 ± 2.5	1.6 ± 2.3
SFA (%)	12.4 ± 2.3	12.4 ± 2.5	12.4 ± 2.4
MUFA (%)	19.8 ± 3.9	19.2 ± 3.6 *	20.3 ± 4.1
PUFA (%)	6.2 ± 2.2	5.8 ± 1.8 *	6.4 ± 2.4
PUFA/SFA	0.5 ± 0.3	0.5 ± 0.2	0.6 ± 0.3
(PUFA + MUFA)/SFA	2.2 ± 0.6	2.1 ± 0.6	2.2 ± 0.7

Results are expressed as the mean ± standard deviation. SFA: saturated fatty acids; MUFA: monounsaturated fatty acids; PUFA: polyunsaturated fatty acids. * *p* ≤ 0.05 (Student’s *t*-test).

**Table 4:** Daily vitamin intake of the total sample and sorted by age groups.

	Total Sample (*n* = 235)	<40 Years Old (*n* = 96)	≥40 Years Old (*n* = 139)
Vitamin B_1_ (mg)	1.4 ± 0.5	1.5 ± 0.6 *	1.3 ± 0.4
Vitamin B_2_ (mg)	1.7 ± 0.6	1.8 ± 0.6 **	1.6 ± 0.5
Vitamin B_3_ (mg)	37.5 ± 9.9	39.8 ± 10.3 **	35.9 ± 9.5
Vitamin B_5_ (mg)	5.3 ± 1.5	5.8 ± 1.6 ***	5.0 ± 1.4
Vitamin B_6_ (µg)	2.2 ± 0.7	2.4 ± 0.7 ***	2.1 ± 0.6
Vitamin B_8_ (µg)	29.7 ± 13.5	31.9 ± 13.6 *	28.2 ± 13.2
Folates (vitamin B_9_) (µg)	280.3 ± 103.6	302.6 ± 105.3 *	264.9 ± 99.9
Vitamin B_12_ (µg)	5.8 ± 4.2	5.4 ± 2.2	6.1 ± 5.1
Vitamin C (mg)	120.8 ± 55.5	120.1 ± 53.3	121.3 ± 57.1
Vitamin A (µg)	1180.8 ± 2510.8	980.8 ± 400.5	1318.9 ± 3245.3
Vitamin D (µg)	3.3 ± 3.2	3.7 ± 3.5	3.1 ± 3.1
Vitamin E (mg)	8.9 ± 4.1	9.1 ± 3.7	8.9 ± 4.4
Vitamin K (µg)	156.5 ± 95.9	172.8 ± 100.9 *	145.2 ± 90.9

Results are expressed as the mean ± standard deviation. * *p* ≤ 0.05, ** *p* ≤ 0.01, and *** *p* ≤ 0.001 indicate significant differences between groups (Student’s *t*-test).

**Table 5:** Daily minerals intake of the total sample and sorted by age group.

	Total Sample (*n* = 235)	<40 Years Old (*n* = 96)	≥40 Years Old (*n* = 139)
Calcium (mg)	800.1 ± 296.9	902.3 ± 352.2 ***	729.5 ± 227.7
Iron (mg)	14.7 ± 4.5	15.6 ± 5.0 *	14.0 ± 3.9
Iodine (µg)	98.9 ± 49.7	104.9 ± 54.9 ***	94.9 ± 45.6
Magnesium (mg)	300.6 ± 92.3	320.6 ± 86.8 *	286.9 ± 93.7
Zinc (mg)	9.7 ± 2.7	10.6 ± 2.7	9.1 ± 2.5
Selenium (µg)	109.8 ± 40.2	114.8 ± 39.9	106.3 ± 40.3
Potassium (mg)	3007.3 ± 829.2	3094.9 ± 749.2	2946.8 ± 877.8
Phosphorus (mg)	1437.9 ± 383.9	1558.6 ± 410.6 ***	1354.5 ± 341.6

Results are expressed as the mean ± standard deviation. * *p* ≤ 0.05 and *** *p* ≤ 0.001 indicate significant differences between groups (Student’s *t*-test).

**Table 6:** Serum homocysteine, serum vitamin B_12_, serum folate, and serum vitamin D concentrations.

	Total Sample (*n* = 235)	<40 Years Old (*n* = 96)	≥40 Years Old (*n* = 139)	Reference Values
Hcy (µmol/L)	11.9 ± 3.1	10.9 ± 2.7 ***	12.6 ± 3.2	4.4–10.8
Vitamin B_12_ (pg/mL)	470.7 ± 176.9	505.3 ± 182.9 *	446.7 ± 169.3	279.0–996.0
Folates (ng/mL)	6.9 ± 3.3	6.7 ± 3.2	7.0 ± 3.4	6.0–20.0
25-Hydroxyvitamin D (ng/mL)	28.9 ± 8.2	28.4 ± 6.7	29.4 ± 9.1	30.0–100.0

Results are expressed as the mean ± standard deviation. * *p* ≤ 0.05 and *** *p* ≤ 0.001 indicate significant differences between groups (Student’s *t*-test).

**Table 7:** Hearing loss in a multiple regression analysis.

Variables	β	SEM	95% CI	*p*-Value
Age (years)	0.588	0.006	0.056 to 0.080	≤0.001
Constant		0.262	−2.074 to −1.042	≤0.001

SEM: standard error of the mean, 95% CI: confidence interval, R = 0.588, R^2^ = 0.346, and R^2^ adjusted = 0.343.

**Table 8:** Hearing loss adjusted by age in a multiple regression analysis.

Variables	β	SEM	95% CI	*p*-Value
Flight hours (h)	0.283	0.000	0.000 to 0.000	≤0.001
Constant		0.108	0.711 to 1.138	≤0.001

SEM: standard error of the mean, 95% CI: confidence interval, R = 0.283, R^2^ = 0.080, and R^2^ adjusted = 0.076.

**Table 9:** Hearing loss adjusted by age in a multiple regression analysis.

Variables	β	SEM	95%CI	*p*-Value
Flight hours (h)	0.246	0.000	0.000 to 0.000	≤0.001
Serum Folate (ng/mL)	−0.143	0.026	−0.107 to −0.005	0.032
Serum Hcy (µmol/L)	0.227	0.028	0.039 to 0.150	≤0.001
Constant		0.432	−0.622 to 1.081	0.596

Hcy: homocysteine, SEM: standard error of the mean, 95% CI: confidence interval, R = 0.419, R^2^ = 0.176, and R^2^ adjusted = 0.165.

## Data Availability

The data presented in this study are available on request from the corresponding author.

## References

[1] Money A., Carder M., Turner S., Hussey L., Agius R. (2011). Surveillance for work-related audiological disease in the UK: 1998–2006. Occupational medicine. Oxf. Engl..

[2] Tak S., Davis R.R., Calvert G.M. (2009). Exposure to hazardous workplace noise and use of hearing protection devices among US workers--NHANES, 1999–2004. Am. J. Ind. Med..

[3] Bhumika N., Prabhu G., Ferreira A., Kulkarni M. (2013). Noise-induced hearing loss still a problem in shipbuilders: A cross-sectional study in goa, India. Ann. Med. Health Sci. Res..

[4] World Health Organization (WHO) Deafness and Hearing Loss. https://www.who.int/news-room/fact-sheets/detail/deafness-and-hearing-loss.

[5] Masterson E.A., Tak S., Themann C.L., Wall D.K., Groenewold M.R., Deddens J.A., Calvert G.M. (2013). Prevalence of hearing loss in the United States by industry. Am. J. Ind. Med..

[6] Roth T.N., Hanebuth D., Probst R. (2011). Prevalence of age-related hearing loss in Europe: A review. Eur. Arch. Oto-Rhino-Laryngol..

[7] Dror A.A., Avraham K.B. (2009). Hearing loss: Mechanisms revealed by genetics and cell biology. Annu. Rev. Genet..

[8] Hong O., Lusk S.L., Ronis D.L. (2005). Ethnic differences in predictors of hearing protection behavior between Black and White workers. Res. Theory Nurs. Pract..

[9] Leensen M.C., van Duivenbooden J.C., Dreschler W.A. (2011). A retrospective analysis of noise-induced hearing loss in the Dutch construction industry. Int. Arch. Occup. Environ. Health.

[10] Davies H., Marion S., Teschke K. (2008). The impact of hearing conservation programs on incidence of noise-induced hearing loss in Canadian workers. Am. J. Ind. Med..

[11] Puga A.M., Pajares M.A., Varela-Moreiras G., Partearroyo T. (2018). Interplay between Nutrition and Hearing Loss: State of Art. Nutrients.

[12] Cheslock M., De Jesus O. (2022). Presbycusis. StatPearls.

[13] Karli R., Gül A., Uğur B. (2013). Effect of vitamin B12 deficiency on otoacoustic emissions. Acta Otorhinolaryngol. Ital..

[14] Lasisi A.O., Fehintola F.A., Yusuf O.B. (2010). Age-related hearing loss, vitamin B12, and folate in the elderly. Otolaryngol.--Head Neck Surg..

[15] Gok U., Halifeoglu I., Canatan H., Yildiz M., Gursu M.F., Gur B. (2004). Comparative analysis of serum homocysteine, folic acid and Vitamin B12 levels in patients with noise-induced hearing loss. Auris Nasus Larynx.

[16] De Luca C., Deeva I., Mariani S., Maiani G., Stancato A., Korkina L. (2009). Monitoring antioxidant defenses and free radical production in space-flight, aviation and railway engine operators, for the prevention and treatment of oxidative stress, immunological impairment, and pre-mature cell aging. Toxicol. Ind. Health.

[17] Irvine D., Davies D.M. (1992). The mortality of British Airways pilots, 1966–1989: A proportional mortality study. Aviat. Space Environ. Med..

[18] Lindgren T., Wieslander G., Dammström B.G., Norbäck D. (2009). Tinnitus among airline pilots: Prevalence and effects of age, flight experience, and other noise. Aviat. Space Environ. Med..

[19] Axelsson A., Sandh A. (1985). Tinnitus in noise-induced hearing loss. Br. J. Audiol..

[20] (2006). Real Decreto 286/2006, de 10 de marzo, sobre la protección de la salud y la seguridad de los trabajadores contra los riesgos relacionados con la exposición al ruido. B.O.E..

[21] Atalay H., Babakurban S., Aydin E. (2016). Evaluation of Hearing Loss in Pilots. Turk Otolarengoloji Ars./Turk. Arch. Otolaryngol..

[22] Pujol R. Journey into the World of Hearing. http://www.cochlea.eu/es.

[23] Houston D.K., Johnson M.A., Nozza R.J., Gunter E.W., Shea K.J., Cutler G.M., Edmonds J.T. (1999). Age-related hearing loss, vitamin B-12, and folate in elderly women. Am. J. Clin. Nutr..

[24] Cadoni G., Agostino S., Scipione S., Galli J. (2004). Low serum folate levels: A risk factor for sudden sensorineural hearing loss?. Acta Oto-Laryngol..

[25] Durga J., Verhoef P., Anteunis L.J., Schouten E., Kok F.J. (2007). Effects of folic acid supplementation on hearing in older adults: A randomized, controlled trial. Ann. Intern. Med..

[26] Jacques P.F., Selhub J., Bostom A.G., Wilson P.W., Rosenberg I.H. (1999). The effect of folic acid fortification on plasma folate and total homocysteine concentrations. New Engl. J. Med..

[27] Martínez-Vega R., Murillo-Cuesta S., Partearroyo T., Varela-Moreiras G., Varela-Nieto I., Pajares M.A. (2016). Long-Term Dietary Folate Deficiency Accelerates Progressive Hearing Loss on CBA/Ca Mice. Front. Aging Neurosci..

[28] Martínez-Vega R., Garrido F., Partearroyo T., Cediel R., Zeisel S.H., Martínez-Álvarez C., Varela-Moreiras G., Varela-Nieto I., Pajares M.A. (2015). Folic acid deficiency induces premature hearing loss through mechanisms involving cochlear oxidative stress and impairment of homocysteine metabolism. FASEB J..

[29] Curhan S.G., Eavey R.D., Wang M., Rimm E.B., Curhan G.C. (2014). Fish and fatty acid consumption and the risk of hearing loss in women. Am. J. Clin. Nutr..

[30] Martínez-Vega R., Partearroyo T., Vallecillo N., Varela-Moreiras G., Pajares M.A., Varela-Nieto I. (2015). Long-term omega-3 fatty acid supplementation prevents expression changes in cochlear homocysteine metabolism and ameliorates progressive hearing loss in C57BL/6J mice. J. Nutr. Biochem..

[31] Asociación Española de Audiología (AEDA) (2002). Normalización de las pruebas audiológicas (I): La audiometría tonal liminar. Auditio.

[32] Salvador Castell G., Serra-Majem L., Ribas-Barba L. (2015). What and how much do we eat? 24-hour dietary recall method. Nutr. Hosp..

[33] European Food Safety Authority (EFSA) (2014). Guidance of the EU Menu methodology. EFSA J..

[34] European Food Safety Authority (EFSA) (2017). Dietary Reference Valuesfor nutrients. Summ. Rep. FSA Support. Publ..

[35] Aranceta Bartrina J., Arija Val V.V., Maíz Aldalur E., Martínez de Victoria Muñoz E., Ortega Anta R.M., Pérez-Rodrigo C., Quiles Izquierdo J., Rodríguez Martín A., Román Viñas B., Salvador Castell G. (2016). Dietary Guidelines for the Spanish population (SENC, diciembre 2016); the new graphic icon of healthy food. Nutr. Hosp..

[36] (2009). Real Decreto 1856/2009, de 4 de diciembre, de procedimiento para el reconocimiento, declaración y calificación del grado de discapacidad, y por el que se modifica el Real Decreto 1971/1999, de 23 de diciembre. B.O.E..

[37] Nair S., Kashyap R.C. (2009). Prevalence of Noise Induced Hearing Loss in Indian Air Force Personnel. Med. J. Armed Forces India.

[38] Al-Omari A.S., Al-Khalaf H.M., Hussien N.F.M. (2018). Association of Flying Time with Hearing Loss in Military Pilots. Saudi J. Med. Med. Sci..

[39] Partearroyo T., Murillo-Cuesta S., Vallecillo N., Bermúdez-Muñoz J.M., Rodríguez-de la Rosa L., Mandruzzato G., Celaya A.M., Zeisel S.H., Pajares M.A., Varela-Moreiras G. (2019). Betaine-homocysteine S-methyltransferase deficiency causes increased susceptibility to noise-induced hearing loss associated with plasma hyperhomocysteinemia. FASEB J..

[40] Copp E.K., Green N.R. (1991). Dietary intake and blood lipid profile survey of fighter pilots at Tyndall Air Force Base. Aviat. Space Environ. Med..

[41] 4Stark A.H., Weis N., Chapnik L., Barenboim E., Reifen R. (2008). Dietary intake of pilots in the Israeli Air Force. Mil. Med..

[42] Daskalopoulos C., Palermos J., Zoga T., Stavropoulos A., KYRIAKOS K. (1993). Correlation of life-style and dietary concomitants of Greek pilots with serum analytes. AGARD Nutr. Metab. Disord. Lifestyle Aircrew 4 p(SEE N 93-32240 12-54).

[43] Ruiz E., Ávila J.M., Valero T., del Pozo S., Rodriguez P., Aranceta-Bartrina J., Gil Á., González-Gross M., Ortega R.M., Serra-Majem L. (2015). Energy Intake, Profile, and Dietary Sources in the Spanish Population: Findings of the ANIBES Study. Nutrients.

[44] Taleghani E.A., Sotoudeh G., Amini K., Araghi M.H., Mohammadi B., Yeganeh H.S. (2014). Comparison of Antioxidant Status between Pilots and Non-flight Staff of the Army Force: Pilots May Need More Vitamin C. Biomed. Environ. Sci..

[45] Tang D., Tran Y., Shekhawat G.S., Burlutsky G., Mitchell P., Gopinath B. (2021). Dietary Fibre Intake and the 10-Year Incidence of Tinnitus in Older Adults. Nutrients.

[46] Gratton M.A., Schulte B.A. (1995). Alterations in microvasculature are associated with atrophy of the stria vascularis in quiet-aged gerbils. Hear. Res..

[47] Partearroyo T., Samaniego-Vaesken Mª L., Ruiz E., Varela-Moreiras G. (2018). Assessment of micronutrients intakes in the Spanish population: A review of the findings from the ANIBES study. Nutr. Hosp..

[48] Celik M., Koyuncu İ. (2018). A Comprehensive Study of Oxidative Stress in Tinnitus Patients. Indian J. Otolaryngol. Head Neck Surg..

[49] Petridou A.I., Zagora E.T., Petridis P., Korres G.S., Gazouli M., Xenelis I., Kyrodimos E., Kontothanasi G., Kaliora A.C. (2019). The Effect of Antioxidant Supplementation in Patients with Tinnitus and Normal Hearing or Hearing Loss: A Randomized, Double-Blind, Placebo Controlled Trial. Nutrients.

[50] Prasad K.N., Bondy S.C. (2020). Increased oxidative stress, inflammation, and glutamate: Potential preventive and therapeutic targets for hearing disorders. Mech. Ageing Dev..

[51] Le Prell C.G., Yamashita D., Minami S.B., Yamasoba T., Miller J.M. (2007). Mechanisms of noise-induced hearing loss indicate multiple methods of prevention. Hear. Res..

